# hUC-EVs-ATO reduce the severity of acute GVHD by resetting inflammatory macrophages toward the M2 phenotype

**DOI:** 10.1186/s13045-022-01315-2

**Published:** 2022-07-21

**Authors:** Yan Su, Xueyan Sun, Xiao Liu, Qingyuan Qu, Liping Yang, Qi Chen, Fengqi Liu, Yueying Li, Qianfei Wang, Bo Huang, Xiao-Hui Huang, Xiao-Jun Zhang

**Affiliations:** 1 Peking University People’s Hospital, Peking University Institute of Hematology, No. 11 Xizhimen South Street, Xicheng District, Beijing, China; 2 Beijing Key Laboratory of Hematopoietic Stem Cell Transplantation, Beijing, China; 3 Collaborative Innovation Center of Hematology, Peking University, Beijing, China; 4 National Clinical Research Center for Hematologic Disease, Beijing, China; 5 CAS Key Laboratory of Genomic and Precision Medicine, Collaborative Innovation Center of Genetics and Development, Beijing Institute of Genomics, Chinese Academy of Sciences, Beijing, China; 6 Chinese Academy of Sciences, China National Center for Bioinformation, Beijing, China; 7 Department of Immunology, Institute of Basic Medical Sciences, Chinese Academy of Medical Sciences, Beijing, China

**Keywords:** hUC-EVs-ATO, Acute graft-versus-host disease, Macrophage, Autophagy

## Abstract

**Background:**

Both extracellular vesicles from mesenchymal stromal cell-derived human umbilical cords (hUC-EVs) and arsenic trioxides (ATOs) have been demonstrated to treat acute graft-versus-host disease (aGVHD) via immunomodulation. Apart from immunomodulation, hUC-EVs have a unique function of drug delivery, which has been proposed to enhance their efficacy. In this study, we first prepared ATO-loaded hUC-EVs (hUC-EVs-ATO) to investigate the therapeutic effect and potential mechanisms of hUC-EVs-ATO in a mouse model of aGVHD after allogeneic hematopoietic stem cell transplantation (HSCT).

**Methods:**

An aGVHD model was established to observe the therapeutic effects of hUC-EVs-ATO on aGVHD. Target organs were harvested for histopathological analysis on day 14 after transplantation. The effects of hUC-EVs-ATO on alloreactive CD4^+^ were evaluated by flow cytometry in vivo and in vitro. Flow cytometry, RT-PCR, immunofluorescence colocalization analysis and Western blot (Wb) analysis were performed to examine macrophage polarization after hUC-EV-ATO treatment. The cytokines in serum were measured by a cytometric bead array (CBA). TEM, confocal microscopy and Wb were performed to observe the level of autophagy in macrophages. A graft-versus-lymphoma (GVL) mouse model was established to observe the role of hUC-EVs-ATO in the GVL effect.

**Results:**

The clinical manifestations and histological scores of aGVHD in the hUC-EVs-ATO group were significantly reduced compared with those in the ATO and hUC-EVs groups. The mice receiving hUC-EVs-ATO lived longer than the control mice. Notably, hUC-EVs-ATO interfering with alloreactive CD4^+^ T cells differentiation were observed in aGVHD mice but not in an in vitro culture system. Additional studies showed that depletion of macrophages blocked the therapeutic effects of hUC-EVs-ATO on aGVHD. Mechanistically, hUC-EVs-ATO induced autophagic flux by inhibiting mammalian target of rapamycin (mTOR) activity to repolarize M1 to M2 macrophages. Additionally, using a murine model of GVL effects, hUC-EVs-ATO were found not only to reduce the severity of aGVHD but also to preserve the GVL effects. Taken together, hUC-EVs-ATO may be promising candidates for aGVHD treatment.

**Conclusions:**

hUC-EVs-ATO enhanced the alleviation of aGVHD severity in mice compared with ATO and hUC-EVs without weakening GVL activity. hUC-EVs-ATO promoted M1 to M2 polarization via the mTOR-autophagy pathway. hUC-EVs-ATO could be a potential therapeutic approach in aGVHD after allo-HSCT.

**Supplementary Information:**

The online version contains supplementary material available at 10.1186/s13045-022-01315-2.

## Background

It has been demonstrated that allogeneic hematopoietic stem cell transplantation (allo-HSCT) is a curative therapy for various hematopoietic malignancies [1, 2]. Acute GVHD (aGVHD), a life-threatening complication after transplantation, occurs in nearly 40–60% of patients following allo-HSCT [3, 4]. Corticosteroids and alternative therapies have been clinically applied; however, the outcome of aGVHD patients is unsatisfactory. The activation of donor T cells and the release of inflammatory cytokines play a fundamental role in the development of aGVHD [5, 6], and the pathophysiology of aGVHD is still only partially understood. Therefore, it is essential to better understand the pathogenesis of aGVHD and develop novel treatment regimens.

The role of macrophages in aGVHD pathogenesis is becoming increasingly apparent [7]. In response to different stimulations, macrophages display great plasticity. IFN-γ- and LPS-primed macrophages (M1, classically activated macrophages) with high expression of CD80, CD86 and iNOS produce many proinflammatory cytokines, including IL-1β, TNF‐α and IL-6 [8, 9]. In contrast, macrophages treated with IL-4 or IL-13 (M2, alternatively activated macrophages) are characterized by CD206, arginase I, FIZZ1 and Ym1 [10]. Similar to M1 macrophages, M2 macrophages secrete many anti-inflammatory cytokines, such as IL-10. A study by Hogenes et al. [11] revealed that macrophage depletion decreased survival and aggravated the histomorphology of the xenogeneic reaction in aGVHD. A recent study implied that the infiltration of activated M1 macrophages was associated with aGVHD severity in the oral mucosa [12]. A higher M1/M2 ratio in grafts was observed to be correlated with the occurrence of grade 2–4 aGVHD [13]. Interestingly, we also found that the number of M1 macrophages increased and M2 macrophages decreased during aGVHD [14], and shifting from M1 to M2 macrophages could be a potential therapeutic target in aGVHD.

Mesenchymal stromal cells (MSCs) have been widely used for immunomodulatory and immune-mediated disorders, including inflammatory bowel disease (IBD), sepsis, multiple sclerosis (MS), GVHD and arthritis [15–18]. Accumulating evidence supports that the immunoregulatory functions of MSCs partly rely on the release of extracellular vesicles (EVs) [19–23]. EVs consist of a group of different types of spherical membrane vesicles containing a variety of bioactive components. They have been demonstrated to have immunosuppressive effects on the phenotype, function, survival and homing of multiple immune cells and play a role in multiple autoimmune and inflammatory diseases [24–26]. Recently, EVs were found to carry exogenous therapeutic agents [27] and could be engulfed by macrophages, which converts the phenotypes of the macrophages [28]. Arsenic trioxide (ATO) is a well-known traditional Chinese drug used to treat acute promyelocytic leukemia (APL) [29, 30]. In recent years, several reports have shown that ATO is a promising treatment for dysregulated immune disease [31–34]. Moreover, ATO could improve the clinical symptoms in mice with sclerodermatous GVHD [35]. ATO was also observed to prolong the survival of aGVHD mice by shifting macrophages toward the M2 status in our previous study [14]. However, its actual use is limited due to the significant toxicity of ATO. If encapsulated in some type of drug vehicle, ATO may be less toxic and more effective; thus, we tried to use EVs from MSCs as a drug carrier for ATO and explored its therapeutic effects on aGVHD mice.

In this study, we aimed to use EVs loaded with ATO (hUC-EVs-ATO) to achieve greater therapeutic and immunosuppressive properties and enhance their activity against aGVHD by targeting macrophage polarization. Additionally, we observed that hUC-EVs-ATO alleviated aGVHD without interfering with the graft-versus-lymphoma (GVL) effects in an allogeneic GVL mouse model.

## Materials and methods

### Preparation and characterization of hUC-EVs-ATO

We obtained human umbilical cord-derived MSCs (hUC-MSCs) from Beijing iCELL Biotechnology Co., Ltd. (Beijing, China). The characteristics of the hUC-MSCs are shown in the supplementary information, Additional file 1: Fig. S1. First, 1 × 10^7^ hUC-MSCs in 5 mL of culture medium were treated with 3 mg of ATO (Sigma-Aldrich, #311383) and then exposed to ultraviolet radiation (UBV, 300 J m^−2^) for 1 h. To obtain EVs primed with ATO, the culture supernatants were collected and centrifuged at 500 × g for 10 min to remove the cells and then centrifuged at 14,000×*g* for 2 min to remove the debris. Finally, the supernatants were further ultracentrifuged at 14,000×*g* for 1 h at 4 °C to pellet the EVs [36]. The pelleted EVs were washed and suspended in sterile phosphate-buffered saline (PBS) to form hUC-EVs-ATO for the following experiments. After lysing the EVs via NP-40 and applying an ultrasound crusher, the concentration of ATO in the EVs was measured by Q-Exactive mass spectrometry. Based on the recommendation of the International Society for Extracellular Vesicles [37, 38], the size distribution, concentration and surface markers (CD29, CD44, CD9 and CD81) of the generated hUC-EVs and hUC-EVs-ATO were characterized by nanoparticle tracking analysis (NTA), transmission electron microscopy (TEM) and flow cytometry.

### Mouse aGVHD models

Male C57BL/6 mice and female BALB/c mice, 6–8 weeks old, were purchased from Charles River Laboratory (Beijing, China). The mice were kept in a specific pathogen-free environment, and all animal experiments were approved by the Ethical Committee of Peking University People’s Hospital.

The mouse aGVHD model was induced based on a previously described method [39]. Before transplantation, recipient BALB/c mice received water containing erythromycin (Solarbio, 250 mg/L) and gentamicin sulfate (Solarbio, 320 mg/L) for 7 days to prevent intestinal infection. BALB/c mice were irradiated with a myeloablative dose of 8 Gy prior to transplantation, after which T cell-depleted bone marrow (BM) cells (5 × 10^6^) supplemented with splenocytes (1 × 10^7^) harvested from donor C57BL/6 mice were infused into recipient BALB/c mice via the tail vein within 4 to 6 h. The aGVHD mice were randomly divided into four groups: One group was treated with PBS alone as a negative control, and the other groups were intraperitoneally injected with ATO (1 mg/kg), hUC-EVs and hUC-EVs-ATO for 5 consecutive days beginning on day 7 post-C57BL/6 cell transfusion. The number of EVs was approximately 1 × 10^6^/mouse, containing 4 nmol ATO. Moreover, we administered clodronate liposomes (Yeasen, Shanghai) by intravenous injection (5 mg/mL, 200 μL, every 4 days) to deplete the mouse macrophages starting on day 5 and then injected the abovementioned drugs after 48 h. The mice at the endpoint were killed on day 14 post-induction to perform the following experiments. All animal studies were conducted according to institutional guidelines.

### Histological and clinical assessment

Target organs (skin, liver and gut) were extracted at the indicated time points (day 14 after transplantation). Tissue samples were fixed in 10% (v/v) formalin at 4 °C overnight and embedded in paraffin. Five-micrometer slices were used for hematoxylin and eosin (H&E) staining. As previously described [40], H&E staining of these target organs was used to evaluate the severity of aGVHD. All slides were observed using a NanoZoomer S360 digital slice scanner (C13220-01). Clinical assessment was implemented from 7 days after infusion of donor cells and was scored on the basis of skin integrity, fur texture, weight loss, posture and activity as previously described [41]. The scoring system denoted 0 as good and 2 as poor for the sum of each parameter, with a total score range of 0–10 points.

### Immunofluorescence

Immunofluorescence staining was performed to detect M1 macrophages (F4/80^+^iNOS^+^ cells) and M2 macrophages (F4/80^+^CD206^+^ cells) in the liver and intestine. Primary antibodies included antibodies against F4/80 (CST, #700766), iNOS (Boster, BA0362) and CD206 (R&D, AF2535). Nuclei were stained with 4′,6-diamidino-2-phenyl-indole (DAPI, abs47047616). Confocal microscopy (TCS-SP8 STED 3X) was used to visualize the slides.

### GVL model and bioluminescence imaging

To assess the GVL effect, 1 × 10^5^ A20 lymphoma cells (A20-luc, H-2d, National Collection of Authenticated Cell Cultures, China) expressing luciferase combined with T cell-depleted BM cells or T cell-depleted BM cells and splenocytes from donor C57/BL6 mice were infused into BALB/c recipients on the day of transplantation. From day 1 to day 5 after transplantation, hUC-EVs-ATO or PBS was given to the mice infused with BM cells, splenocytes and A20-luc. On days 7, 14 and 21 after transplantation, the mice were intraperitoneally injected with 200 μg firefly luciferin to evaluate the tumor burden with the Xenogen IVIS 100 Bioluminescent Imaging System (Caliper Life Sciences, Hopkinton, MA).

### Generation and stimulation of macrophages

Bone marrow-derived macrophages (BMDMs) were collected from the femurs and tibia of 7-week-old C57BL/6 mice and cultured in RPMI-1640 medium containing 10% FBS (Gibco, 10099141), 50 ng/ml M-CSF (PeproTech, 315-02-10) and 1% penicillin–streptomycin (Gibco, PS2004HY) at 37 °C in 5% CO_2_ for 7 days. Microscopic observation and flow cytometry with PE/cyanine7-conjugated anti-F4/80 (BioLegend, 123114) and BV421-conjugated anti-CD11b (BioLegend, 101236) were used to identify the BMDMs. On the sixth day, 100 ng/mL LPS (Sigma-Aldrich, L4391) and 20 ng/mL IFN-γ (PeproTech, 500-P119-50) or 20 ng/ml IL-4 (PeproTech, 214-14) were added to induce BMDM-M1 or BMDM-M2 macrophages. Twenty-four hours later, the stimulated macrophages were treated with EV, ATO or EV-ATO for 24 h.

The RAW264.7 murine macrophage cell line was purchased from Zhong Qiao Xin Zhou Biotechnology (Shanghai, China) and cultured in RPMI-1640 medium containing 10% FBS and 1% penicillin–streptomycin. The induction of polarization and the intervention was identical to that described above.

Peritoneal macrophages were harvested from mice as reported previously [42]. Briefly, mice were injected intraperitoneally with 5 ml of ice-cold PBS (Solarbio, China). The mouse ascites was collected and centrifuged (350 g for 5 min at 4°) to obtain the cell pellets, which were then resuspended in incomplete medium. These cells were incubated in Petri plates for 3 h (5% CO_2_, 37 °C), unattached cells were discarded, and adherent cells were obtained. The identification of peritoneal macrophages was identical to that of BMDMs (Additional file 1: Fig. S2).

### Quantitative real-time RT-PCR

Total RNA was obtained with TRIzol reagent (Thermo Fisher, USA, 15596026), and cDNA was synthesized from RNA using a reverse transcription kit (Takara, RR047A). Real-time PCR was performed using Power SYBR Green RT-PCR Reagent (Takara, RR820A) with specific primers based on the manufacturer’s instructions. GAPDH was used as an endogenous reference gene in each reaction, and all of the samples were assessed in triplicate. The PCR primer sequences are listed in the supplement (Additional file 2: Table S1).

#### Polarization conditions for T cells

CD4^+^ T cells were immunomagnetically isolated from the spleens of wild-type mice. For induction of Th1 differentiation, we cultured CD4^+^ T cells with anti-CD3/CD28 antibody (Dynabeads Mouse T activator, Life Technologies, Carlsbad, CA), IL-12 (10 ng/ml, R&D Systems) and anti-IL-4 (10 µg/ml, Bio X Cell) for 72 h. For induction of Th17 differentiation, we cultured CD4^+^ T cells with anti-CD3/CD28 antibody (Dynabeads Mouse T activator), IL-6 (10 ng/ml, R&D Systems), TGF-β (2 ng/ml, R&D Systems), anti-IL-2(10 µg/ml, Bio X Cell), anti-IL-4 (10 µg/ml, Bio X Cell) and anti-IFN-γ (10 µg/ml, R&D Systems) for 72 h. For induction of Treg differentiation, we cultured CD4^+^ T cells with anti-CD3/CD28 antibody (Dynabeads Mouse T activator), TGF-β (5 ng/ml, R&D Systems), IL-2 (10 ng/ml, Bio X Cell), anti-IL-4 (10 µg/ml, Bio X Cell) and anti-IFN-γ (10 µg/ml, R&D Systems) for 72 h. Flow cytometry was used for the differentiation and activation of CD4^+^ T cells.

### Flow cytometry

The phenotype of the macrophages was identified by flow cytometry staining with PE/cyanine7-conjugated anti-F4/80 (BioLegend, 123114), BV421-conjugated anti-CD11b (BioLegend, 101236), PE-conjugated anti-CD86 (BioLegend, 123114) and APC-conjugated anti-CD206 (BioLegend, 123114). It should be noted that macrophages were preincubated with anti-mouse CD16/32 (BioLegend, 101320) to block Fc receptors for 5 min at room temperature before staining. For activation and differentiation of CD4+ T cells, flow cytometry staining with AF700-conjugated anti-CD45 (BioLegend, 109822), BV510-conjugated anti-CD3 (BioLegend, 100233), FITC-conjugated anti-CD4 (BioLegend, 100406), APC-conjugated anti-CD69 (BioLegend, 104514), APC-conjugated anti-IL17A (BioLegend, 506916), PE-conjugated anti-IFN-γ (BioLegend, 113604), APC-conjugated anti-CD25 (BD, 557192) and PE-conjugated anti-foxp3 (eBioscience, 12-5773-82) was used. Samples were washed in FACS buffer and stained with the corresponding antibodies for surface marker analysis. For intracellular cytokine staining, the cells were fixed and permeabilized as described in the manufacturer’s instructions (eBioscience, 00-5523-00). Data were analyzed using FlowJo software.

### Western blot analysis

Cells were harvested and lysed with RIPA lysis buffer and protease inhibitor on ice, and 20–40 μg of cell lysates were electrophoresed on 8% and 12% SDS–polyacrylamide gels and then transferred to polyvinylidene fluoride membranes. Membranes were blocked in 5% skim milk (Solarbio) for 1 h at room temperature, incubated with primary antibodies, including anti-phospho(p)-mTOR (Cell Signaling Technology, 5536 T), anti-mTOR (Cell Signaling Technology, 2983 T), anti-LC3B (Cell Signaling Technology, 12741 T), anti-SQSTM1/p62 (Cell Signaling Technology, 5114 T), anti-Arg1 (Cell Signaling Technology, 93668 T) and anti-GAPDH (Biosharp, BL006B), for 1 h at room temperature, and then probed with goat anti-mouse IgG secondary antibody (1:5000, Abcam) for 1 h at room temperature. Membranes were visualized using a chemiluminescence kit (Applygen, P1010-100) and analyzed by ImageJ.

### Transmission electron microscopy

Cells were seeded and treated in 6-well plates. After 48 h, 2.5% glutaraldehyde, 1% osmium tetroxide, an increasing gradient of ethanol and acetone and Spurr’s resin were used to fix, dehydrate and embed the cells, respectively. After slicing with an electron microscope (EM) UC6 ultramicrotome (Leica Microsystems, Wetzlar, Germany), the samples were adhered to uncoated copper grids and stained with 4% uranyl acetate. Transmission electron microscopy (JEM 1400 PLUS, JEOL, Japan) was used to observe the samples.

### GFP-mCherry-LC3B Transfection

RAW264.7 cells were transfected with the GFP-mCherry-LC3B fusion protein following the manufacturer’s instructions (GeneChem Co., Ltd., Shanghai, China). In brief, the cells were transduced at 70–80% confluence and treated with hUC-EVs-ATO. The sample was observed and imaged using a TCS-SP8 confocal laser scanning microscope (Leica, Germany).

### Statistical analysis

All data in our study were derived from at least three independent experiments, and each experiment was conducted in triplicate. Data were processed with GraphPad Prism 7 and were described as the means ± SD. Unpaired Student’s t test and one-way analysis of variance were applied to analyze the difference between two or multiple groups. The log-rank test was performed to compare survival. Statistical significance was considered at *P* < 0.05.

## Results

### Characterization of hUC-EVs-ATO

Using NTA, the size distributions of the hUC-EVs and hUC-EVs-ATO both demonstrated a bell-shaped curve ranging from 100 to 1000 nm (Additional file 1: Fig. S3A). Morphological features using TEM showed round vesicles in both the hUC-EVs and hUC-EVs-ATO (Additional file 1: Fig. S3B). High expression of the transmembrane proteins CD9, CD81, CD44 and CD29, which are classical common surface markers on EVs, was observed on hUC-EVs and hUC-EVs-ATO by flow cytometry (Additional file 1: Fig. S3C). No differences between hUC-EVs and hUC-EVs-ATO were noted with regard to their size, morphology or markers.

### Therapeutic administration of hUC-EVs-ATO alleviated aGVHD in a mouse model

**Fig. 1:**
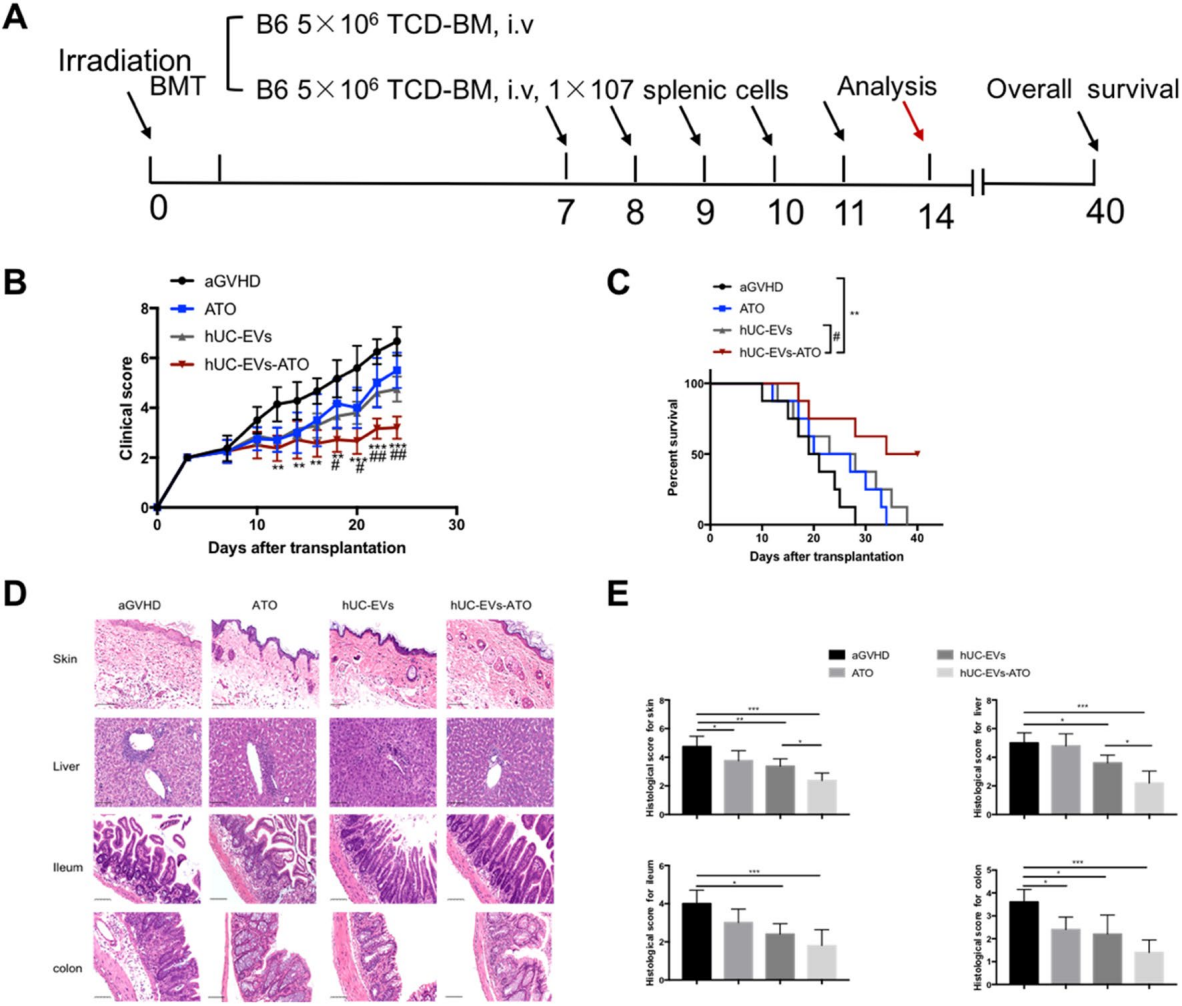
Therapeutic administration of hUC-EVs-ATO alleviated aGVHD in a mouse model. **A** Experimental protocol of BMT mice or aGVHD mice. PBS, ATO, hUC-EVs or hUC-EVs-ATO were intraperitoneally injected into aGVHD mice for 5 consecutive days from day 7 post-transfusion. Data were collected on day 14. **B** Clinical scores of aGVHD mice in each group every 2 days from day 7 after transplantation. **C** Survival of aGVHD mice in each group. **D** Typical images of H&E staining of the skin, liver, ileum and colon of aGVHD mice in each group (magnification × 200). Scale bar, 100 μm. **E** Histological scores of the skin, liver, ileum and colon of aGVHD mice in each group. Data are shown as the mean ± SEM. *P < 0.05; **P < 0.01; ***P < 0.001, * represents a comparison between the hUC-EVs-ATO and aGVHD groups. ^#^*P* < 0.05; ^##^*P* < 0.01; ^###^*P* < 0.001, ^#^ represents a comparison between the hUC-EVs-ATO and hUC-EVs groups. n.s. = not significant. There were six mice in each group

To further investigate whether hUC-EVs-ATO could alleviate ongoing aGVHD, we therapeutically treated aGVHD mice from days 7 to 11 after allo-HSCT. Fourteen days after transplantation, five mice were humanely euthanized from each group, and the target organs (skin, liver and gut) were extracted to perform histological analysis. Mice treated with hUC-EVs and ATO developed similar clinical scores of aGVHD. Significantly, hUC-EVs-ATO showed the lowest clinical scores, and all of these treatment groups showed amelioration of aGVHD severity to some extent (Fig. 1B). Accordingly, pathological examination of the target organs was implemented. Histological changes in each organ coincided with the above results. The aGVHD-associated pathological lesions were the smallest in the group with hUC-EVs-ATO (Fig. 1D, E). Meanwhile, the survival of aGVHD mice infused with hUC-EVs, ATO and hUC-EVs-ATO was prolonged. Specifically, their survival was further enhanced by administering hUC-EVs-ATO compared with that of mice receiving ATO or hUC-EVs (Fig. 1C). All of these data suggested that therapeutic administration of hUC-EVs-ATO alleviated the severity of aGVHD in allogenic HSCT.

### hUC-EVs-ATO modulated T cell populations in mice with aGVHD but not in vitro

**Fig. 2:**
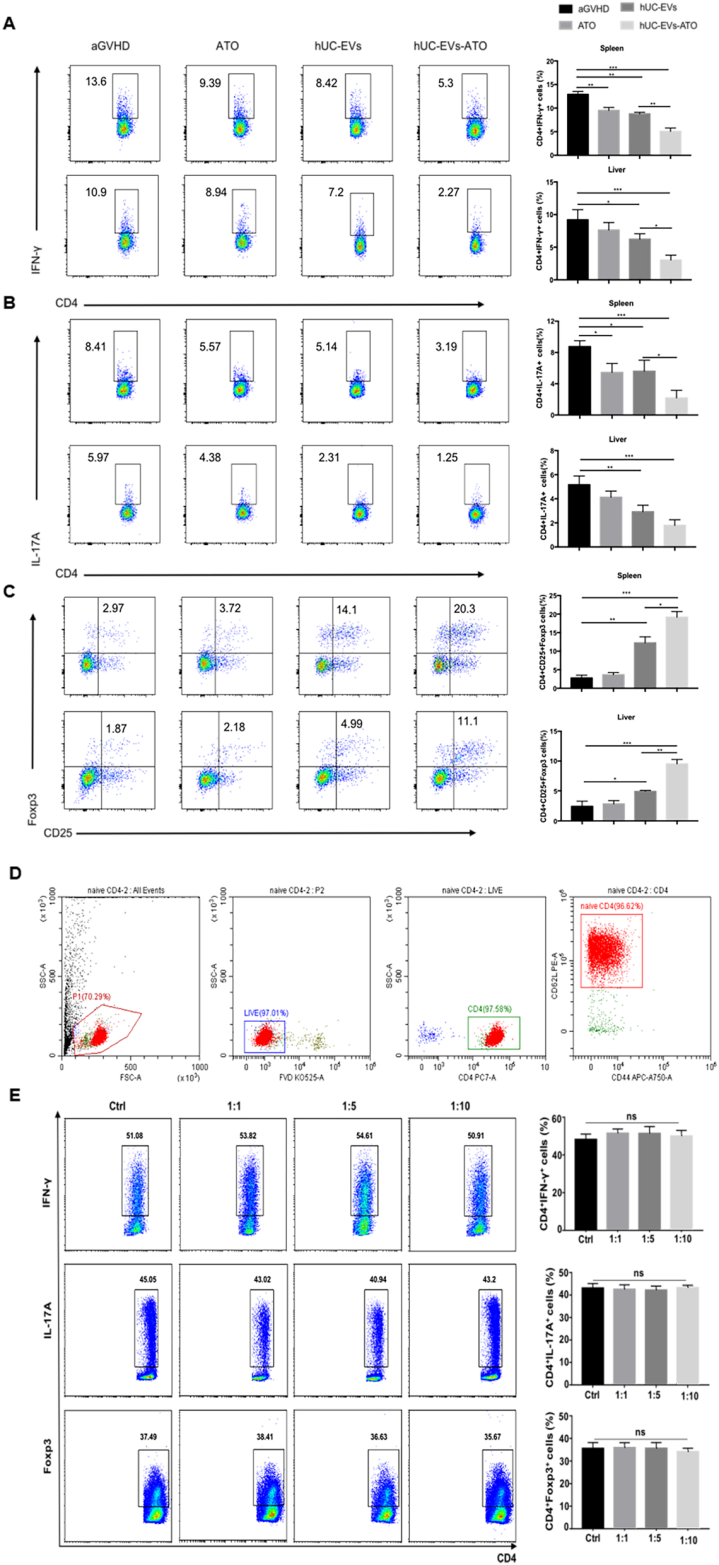
hUC-EVs-ATO modulated T cell populations in mice with aGVHD but not in vitro. On day 14 after transplantation, the frequencies of IFN-γ^+^CD4^+^ (**A**), IL-17^+^CD4^+^ (**B**) and Tregs (**C**) in the liver and spleen of aGVHD mice treated with PBS, ATO, hUC-EVs or hUC-EVs-ATO were tested by flow cytometry. Data are the means ± SEM of six mice per group. (**D**) CD4^+^ T cells were immunomagnetically sorted from the spleens of C57BL/6 mice. The purity of CD4^+^ T cells was measured by flow cytometry. (**E**) After treatment with different doses of hUC-EVs-ATO (CD4^+^ T cells: hUC-EVs-ATO = 1:1, 1:5, 1:10) for 72 h, the frequencies of IFN-γ^+^CD4^+^, IL-17^+^CD4^+^ and Tregs were measured via flow cytometry. The data are representative of three independent experiments. Data are shown as the mean ± SEM. *P < 0.05; **P < 0.01; ***P < 0.001, n.s. = not significant

**Fig. 3:**
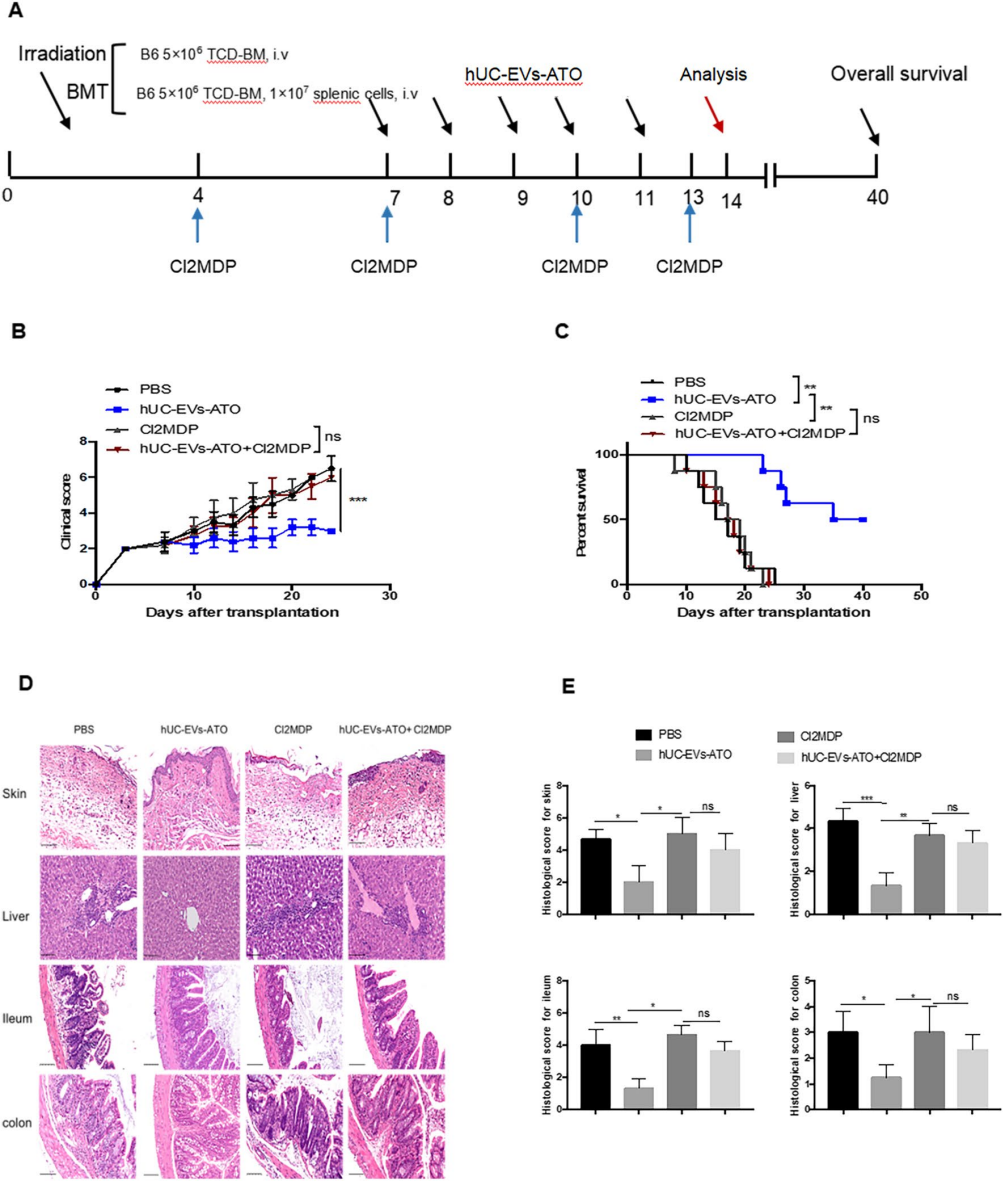
The therapeutic effects of hUC-EVs-ATO are macrophage dependent. **A** Experimental protocol of BMT mice or aGVHD mice. hUC-EVs-ATO were intraperitoneally injected into aGVHD mice for 5 consecutive days from day 7 post-transfusion. Cl2MDP liposomes were intravenously injected into mice 72 h before injecting hUC-EVs-ATO and once every 3 days until day 14 after transplantation, when the mice were killed. Data were collected on day 14. **B** Clinical scores of aGVHD mice in each group every 2 days from day 7 after transplantation. **C** Survival of aGVHD mice in each group. **D** Typical images of H&E staining of the skin, liver, ileum and colon of aGVHD mice in each group (magnification × 200). Scale bar, 100 μm. **E** Histological scores of the skin, liver, ileum and colon of aGVHD mice in each group. Data are shown as the mean ± SEM. *P < 0.05; **P < 0.01; ***P < 0.001, n.s. = not significant. There were six mice in each group

To clarify the possible mechanism by which hUC-EVs-ATO exert an effect on the murine aGVHD model, CD4^+^ T cell subsets in the spleen and liver were tested at day 14. A significantly higher proportion of CD4^+^CD25^+^FoxP3^+^ Tregs but significantly lower frequencies of IFN-γ^+^CD4^+^ and IL-17^+^CD4^+^ T cells were observed in hUC-EVs-ATO-treated mice (Fig. 2A–C). We next isolated highly purified naive CD4^+^ T cells with immunomagnetic beads from the spleens of C57BL/6 mice (Fig. 3D). Unexpectedly, hUC-EVs-ATO neither decreased Th1 and Th17 cells nor increased Treg cells under polarizing conditions (Fig. 2E). Thus, the impact of hUC-EVs-ATO on the alloreactive T cell response was apparent in aGVHD mice but not in an in vitro culture system. Based on these results, we hypothesized that hUC-EVs-ATO indirectly regulated T cell differentiation.

### Therapeutic effects of hUC-EVs-ATO were macrophage dependent

Considering that in vivo T cell differentiation requires macrophages, we administered Cl2MDP liposomes to mice 72 h before injecting hUC-EVs-ATO and again once every 3 days to further determine the role of macrophages in hUC-EVs-ATO therapy. After depletion of macrophages with Cl2MDP liposomes (Additional file 1: Fig. S4), hUC-EVs-ATO did not alleviate aGVHD severity, with no significant differences in clinical scores (Fig. 3B), histopathological scoring (Fig. 3D, E) or survival (Fig. 3C) between the PBS-treated group and the Cl2MDP-treated group. Collectively, our results suggested that depletion of macrophages significantly impaired the benefits of hUC-EVs-ATO in aGVHD mice, which indicated that macrophages were required in hUC-EVs-ATO therapy.

### hUC-EVs-ATO upregulated M2 macrophages in aGVHD mice

**Fig. 4:**
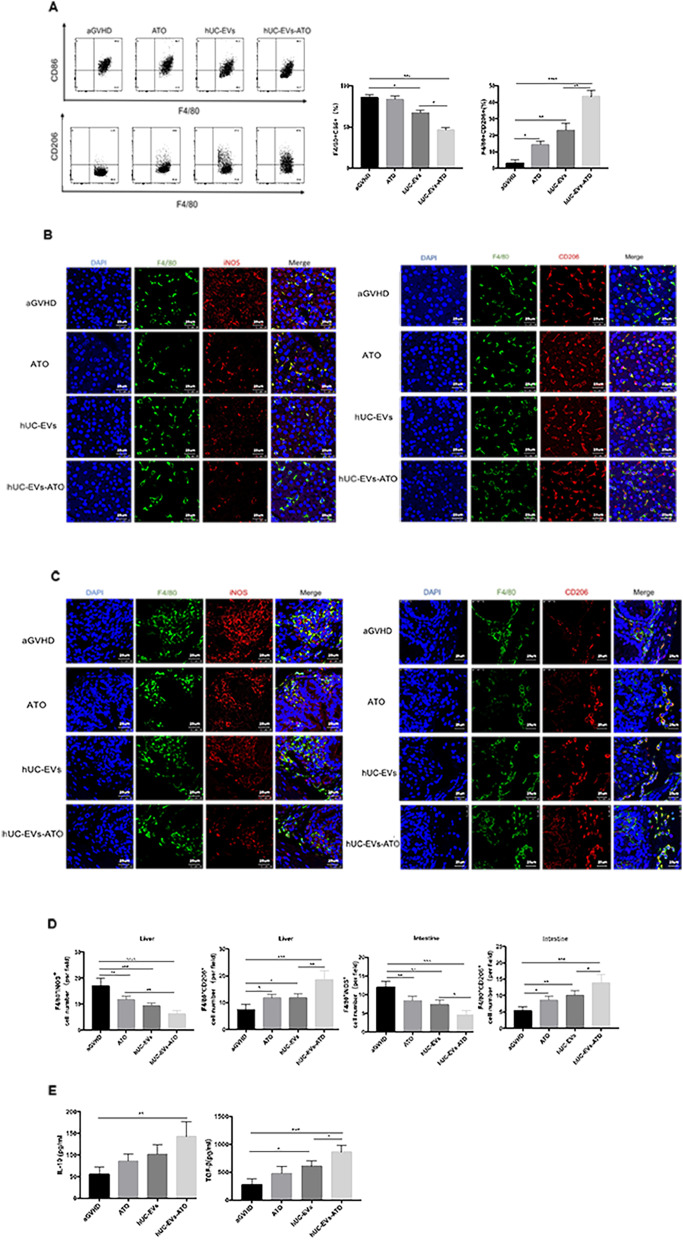
hUC-EVs-ATO upregulated M2 macrophages in aGVHD mice. **A** On day 14 after transplantation, peritoneal macrophages were harvested from the mice and analyzed by flow cytometry. The frequencies of M1 (F4/80^+^CD86^+^) and M2 (F4/80^+^ CD206^+^) macrophages in the peritoneal fluid of mice that received PBS, ATO, hUC-EVs or hUC-EVs-ATO are presented as the mean ± SEM. *P < 0.05; **P < 0.01; ***P < 0.001, n.s. = not significant. There were six mice in each group. **B** On day 14 after transplantation, immunofluorescence images of M1 (F4/80^+^ iNOS^+^) and M2 (F4/80^+^ CD206^+^) macrophages in liver biopsy tissues and **C** intestinal biopsy tissues were obtained in mice that received PBS, ATO, hUC-EVs or hUC-EVs-ATO. Original magnification: × 400. Scale bar, 25 μm. **D** The number of M1 (F4/80^+^iNOS^+^) and M2 (F4/80^+^CD206^+^) macrophages in liver or intestinal biopsy tissues was quantified using a minimum of three mice in each group, with a minimum of six nonoverlapping fields of view per slide. Data represent the mean cell number per square millimeter ± SEM. **E** With CBA, the levels of TNF-α, IL-6, IL-1β, IL-10 and TGF-β in the serum of recipient mice in each group on day 14 after transplantation are described as the mean ± SEM. *P < 0.05; **P < 0.01, ***P < 0.001, n.s. = not significant. There were six mice in each group

To further investigate whether administering hUC-EVs-ATO affects the polarization of macrophages in aGVHD mice, six mice from each group were euthanized at day 14 after transplantation. Peritoneal macrophages were collected from the mice and examined with flow cytometry. The frequency of CD11b^+^ F4/80^+^ CD86^+^ cells representing M1 macrophages was dramatically decreased in mice receiving hUC-EVs, which was further diminished by hUC-EVs-ATO treatment (Fig. 4A). Conversely, the percentage of CD11b^+^ F4/80^+^ CD206^+^ cells representing M2 macrophages showed a large increase in mice receiving additional hUC-EVs, ATO or hUC-EVs-ATO, especially in the hUC-EVs-ATO group (Fig. 4A). The changes in the macrophage phenotype in the liver (Fig. 4B) and intestine (Fig. 4C) observed by immunofluorescence staining were in agreement with the analysis of peritoneal macrophages via flow cytometry (Fig. 4D). Furthermore, M1-related cytokines (TNF-α, IL-6 and IL-1β) as well as M2-related cytokines (IL-10 and TGF-β) in the serum of recipient mice were assessed. The levels of TNF-α, IL-6 and IL-1β in recipient mice were significantly reduced after treatment with hUC-EVs-ATO, while the levels of IL-10 and TGF-β were significantly increased (Fig. 4E). All of these results indicated that the infusion of hUC-EVs-ATO markedly promoted M2 polarization in aGVHD mice.

### hUC-EVs-ATO more effectively polarized M1 macrophages toward M2 macrophages in vitro

**Fig. 5:**
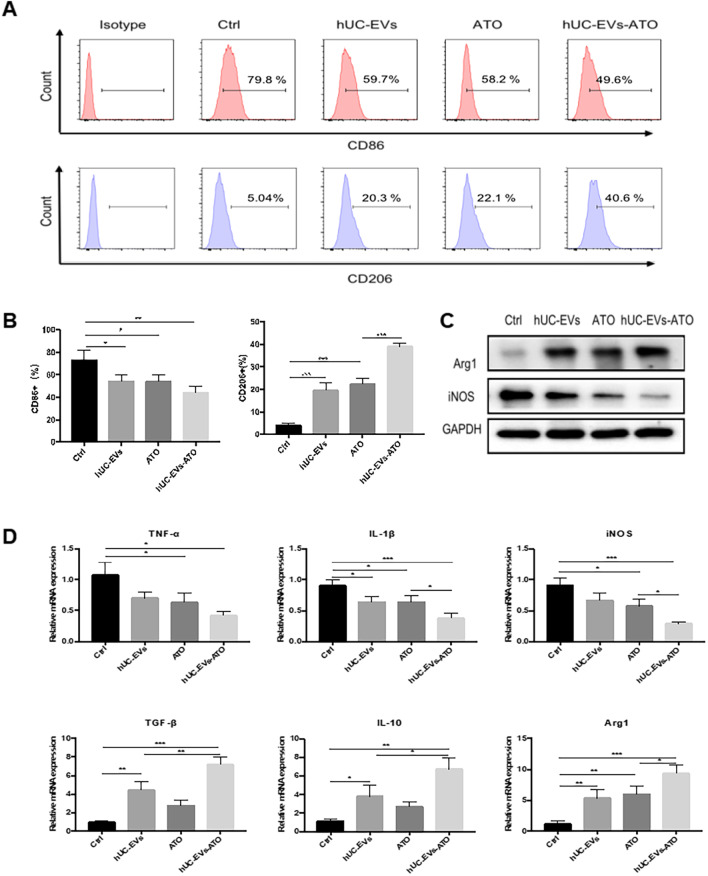
hUC-EVs-ATO more effectively polarized M1 macrophages toward M2 macrophages in vitro. Bone marrow-derived macrophages (BMDMs) were collected from the femurs and tibias of 7-week-old C57BL/6 mice. On the sixth day, 100 ng/mL LPS (Sigma-Aldrich) and 20 ng/mL IFN-γ (PeproTech) were added to induce BMDM-M1. Twenty-four hours later, the stimulated macrophages were treated with hUC-EVs, ATO or hUC-EVs-ATO for 24 h. **A** Changes in the expression of M1- or M2-related markers in BMDM-M1 macrophages observed via flow cytometry after treatment with ATO, hUC-EVs or hUC-EVs-ATO. **B** Frequencies of M1 (CD86^+^) and M2 (CD206^+^) in BMDM-M1 macrophages analyzed by flow cytometry after treatment with ATO, hUC-EVs or hUC-EVs-ATO. **C** Protein expression of Arg1 and iNOS in the cell lysates of BMDM-M1 macrophages with different interferences was detected by immunoblotting analysis. **D** The fold changes at the mRNA level of TNF-α, IL-1β, iNOS, IL-10, TGF-β and Arg1 relative to BMDM-M1 macrophages without any intervention were calculated by the 2^−▲▲^ CT method. The data shown are representative of three independent experiments and are presented as the mean ± SEM. *P < 0.05; **P < 0.01; ***P < 0.001, n.s. = not significant

To further verify whether hUC-EVs-ATO could promote macrophage repolarization from the M1 phenotype toward the M2 phenotype, LPS-conditioned BMDM-M1 macrophages were treated with hUC-EVs, ATO or hUC-EVs-ATO. As expected, the hUC-EVs-ATO-treated group showed a lower number of CD86^+^ M1 macrophages and a higher number of CD206^+^ M2 macrophages than the group treated with hUC-EVs or ATO (Fig. 5A, B). Moreover, the expression of M1-related genes, including IL-1β, TNFα and iNOS, was remarkably downregulated, and the expression of Arg1 and other M2-related genes, including IL-10 and TGF-β, was upregulated in hUC-EVs, ATO and hUC-EVs-ATO (Fig. 5D). Similar expression changes in the protein levels of Arg1 and iNOS were also observed (Fig. 5C). More importantly, this effect was more notable by hUC-EVs-ATO, which indicated that hUC-EVs-ATO could more effectively polarize M1 macrophages into M2 macrophages. Using fluorescence staining, we also found that hUC-EVs-ATO could be taken up by RAW264.7 mouse macrophages, and repolarization effects of hUC-EVs-ATO were also observed in RAW264.7 mouse macrophages (Additional file 1: Fig. S5), further indicating that hUC-EVs-ATO could work through macrophages.

### hUC-EVs-ATO promoted M2 macrophage polarization by inducing autophagy in an mTOR manner

**Fig. 6:**
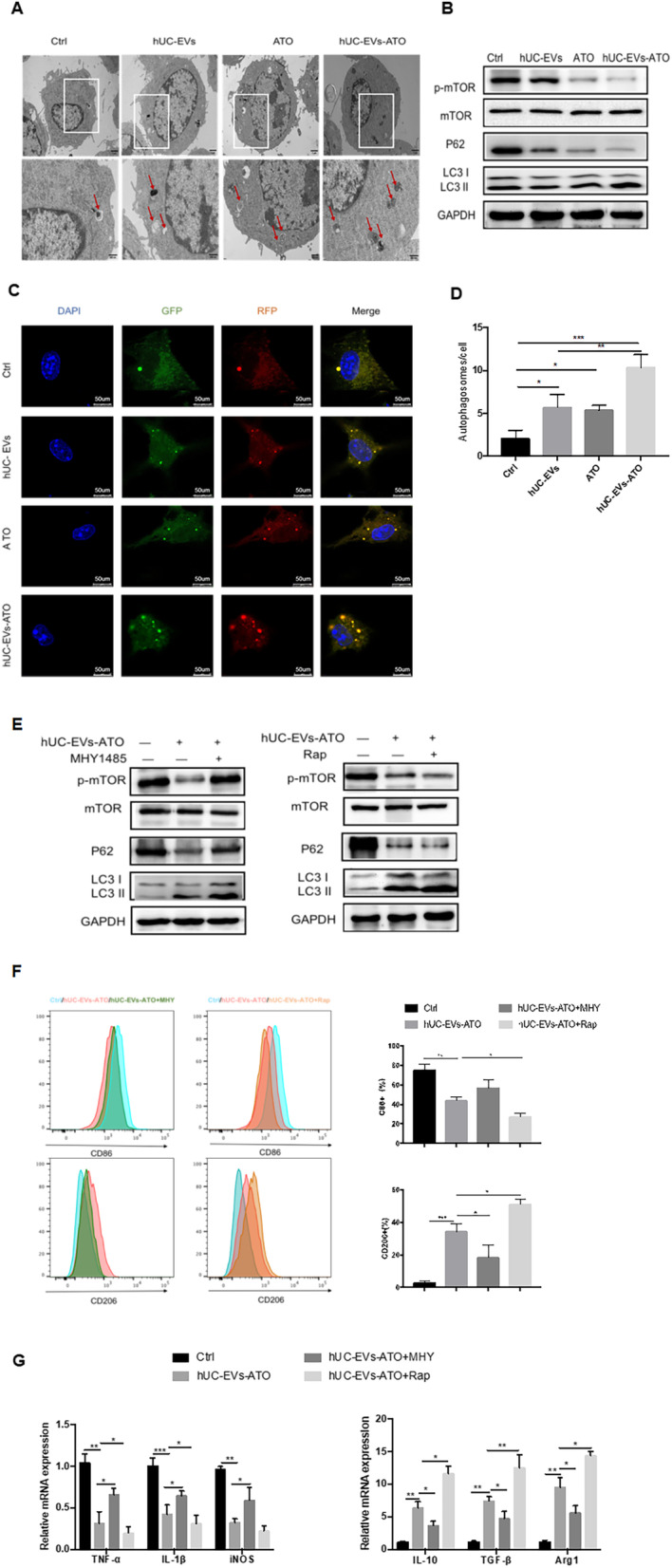
hUC-EVs-ATO promoted M2 macrophage polarization by inducing autophagy in an mTOR-dependent manner. BMDM-M1 macrophages were obtained, and the corresponding intervention methods were identical to those above. **A** Electron micrography was performed on autophagic vesicles of unstimulated BMDM-M1 and BMDM-M1 macrophages stimulated with hUC-EVs, ATO or hUC-EVs-ATO for 24 h. Autophagosomes and autophagic lysosomes are indicated with red arrows. **B** The expression of p-mTOR, mTOR, P62, LC3 I and LC3 II was assayed in the cell lysates of BMDM-M1 macrophages with different interventions by Western blotting. **C** RAW264.7 cells were transfected with GFP-mCherry-LC3B fusion protein, and the number of autophagosomes in macrophages with different interventions was observed with confocal microscopy. Magnification: × 200. Scale bar, 50 μm. Autophagosomes presented with a yellow signal. **D** Number of autophagosomes in each macrophage with different interventions. The data shown are representative of three independent experiments and are presented as the mean ± SEM. *P < 0.05; **P < 0.01, ***P < 0.001, n.s. = not significant. **E** MHY1485, an mTOR activator, or rapamycin, an mTOR inhibitor, was used to treat BMDM-M1 macrophages before hUC-EV-ATO stimulation. The expression of p-mTOR, mTOR, P62, LC3 I and LC3 II was assayed via Western blotting. **F** Changes in the expression of M1- or M2-related markers in BMDM-M1 macrophages and the frequencies of M1 (CD86^+^) and M2 (CD206^+^) in BMDM-M1 macrophages observed via flow cytometry after treatment with hUC-EVs-ATO. **G** The fold changes at the mRNA levels of TNF-α, IL-1β, iNOS, IL-10, TGF-β and Arg1 relative to BMDM-M1 macrophages without any intervention were calculated by the 2^−▲▲^CT method. The data shown are representative of three independent experiments and are presented as the mean ± SEM. *P < 0.05; **P < 0.01; ***P < 0.001, n.s. = not significant

As autophagy has been demonstrated to affect macrophage polarization, we next focused on the effect of hUC-EVs-ATO on autophagy in macrophages. After stimulation with hUC-EVs, ATO or hUC-EVs-ATO for 24 h, BMDM-M1 macrophages showed an accumulation of LC3 II and degradation of the autophagy substrate p62 (Fig. 6B), and an increased number of autophagosomes and LC3 puncture were also observed by electron micrography and confocal microscopy, respectively (Fig. 6A, C). Remarkably, the autophagy levels were the highest in the hUC-EVs-ATO-treated group (Fig. 6C, D).

As activation of mammalian target of rapamycin (mTOR) via phosphorylation has been demonstrated to suppress autophagy, we next paid attention to the impact of hUC-EVs-ATO on mTOR expression. Our data showed that the level of phospho-mTOR (p-mTOR) was reduced after treatment with hUC-EVs-ATO (Fig. 6B). The same trends were observed in peritoneal macrophages derived from hUC-EVs-ATO-treated aGVHD mice (Additional file 1: Fig. S6). To further clarify the role of mTOR in the regulation of the macrophage phenotype, we used an mTOR activator, MHY1485, to treat BMDM-M1 macrophages before hUC-EVs-ATO stimulation. Upon MHY1485 exposure, hUC-EVs-ATO-mediated activation of LC3II expression and inhibition of p-mTOR and P62 were strongly decreased (Fig. 6E). Interestingly, with the upregulation of p-mTOR, the percentages of CD86-positive cells and proinflammatory cytokines (IL-1β, IL-6, and TNF-α), which were inhibited by hUC-EVs-ATO, were significantly increased (Fig. 6F, G). Moreover, the increase in the percentages of CD206-positive and anti-inflammatory cytokines (IL-10 and TGF-β) was strongly reduced (Fig. 6F, G). However, the promoting effects of hUC-EVs-ATO on M2 macrophage polarization were strengthened when macrophages were stimulated with rapamycin, an mTOR inhibitor (Fig. 6E–G). Altogether, these results revealed that hUC-EVs-ATO possibly affected mTOR-dependent autophagy to promote the M1 switch to M2 macrophages.

### hUC-EVs-ATO preserved GVL effects after BMT

**Fig. 7:**
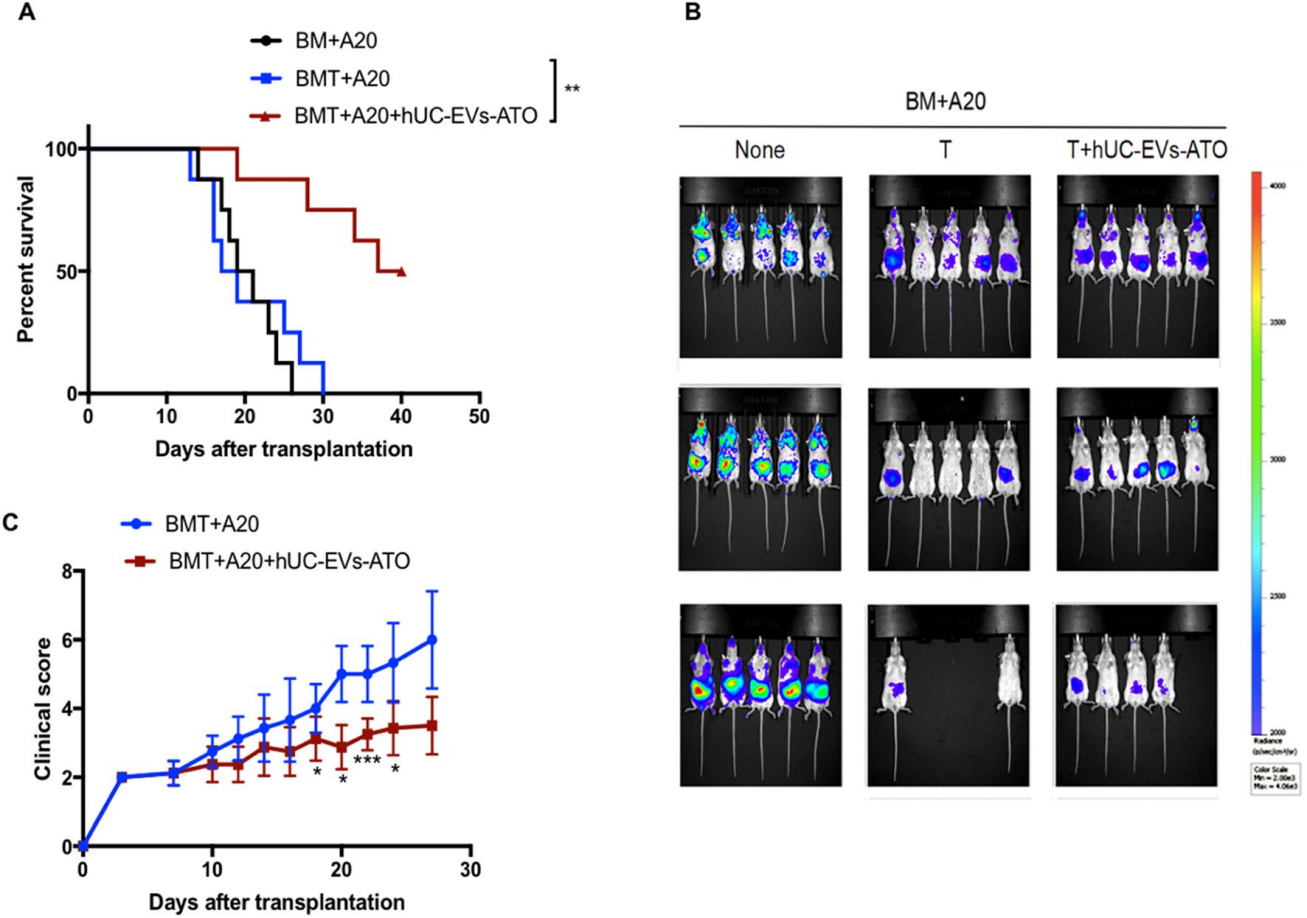
hUC-EVs-ATO preserved GVL effects. A20 lymphoma cells (1 × 10^5^, A20-luc, H-2d) expressing luciferase were infused into BALB/c recipients on the day of transplantation. From day 1 to day 5 after transplantation, hUC-EVs-ATO or PBS was administered to mice. On days 7, 14 and 21 after transplantation, mice were intraperitoneally injected with 200 μg of firefly luciferin and then imaged with a Xenogen IVIS 100 Bioluminescent Imaging System. **A** Survival of mice in each group (*n* = 8/group). **B** Images of tumor growth in recipients exhibiting bioluminescence. **C** Clinical scores of mice in each group (*n* = 8/group)

Preserving the GVL effect is essential for preventing hematological malignancy relapse. To evaluate the impact of hUC-EVs-ATO on GVL activity, A20 murine B lymphoma cells transduced with luciferase (A20-luc) were supplemented with an HSC graft for BLI tumor tracking. All mice transplanted with BM and A20 cells succumbed to the tumors within 26 days. In vivo injection of spleen T cells lacked a tumor BLI signal regardless of the treatment (Fig. 7B), and the mice without hUC-EVs-ATO eventually died from severe GVHD. Remarkably, the mice that received T cells and hUC-EVs-ATO exhibited significantly prolonged survival compared with mice transplanted with BM and A20 cells alone (Fig. 7A). Therefore, these findings indicate that hUC-EVs-ATO treatment alleviated aGVHD and did not compromise GVL activity after allogeneic BMT.

## Discussion

ATO has been found to have an immunoregulatory effect on multiple immune cells [43, 44], and recent studies have reported that it could ameliorate aGVHD [14, 35, 45]. However, the bioavailability of ATO is low due to its rapid plasma clearance and failure to reach specific sites to function [46]. Meanwhile, elevating serum concentrations of ATO by increasing application doses is also difficult to achieve because of the systemic toxicity of ATO. All of these factors limit the efficacy of ATO when it is used alone. EVs derived from MSCs are a heterogeneous membrane vesicle population that contains various types of biological molecules, such as proteins, lipids and microRNAs, and it has been demonstrated that hUC-EVs possess comparable therapeutic activities from which they originate while negating the tumorigenicity and embolism related to MSCs [24–26]. EVs derived from MSCs have been found to prevent aGVHD following allo-HSCT by decreasing the number of cytotoxic T cells and the proinflammatory factors IL-2, TNF-α and IFN-γ while increasing the production of IL-10, leading to less severe symptoms and pathological changes in aGVHD mice [21, 47]. Apart from the function of immunoregulation, hUC-EVs have been found to become drug carriers to deliver their contents through interreceptor–ligand interactions or direct endocytosis [48]. The phospholipid bilayer of hUC-EVs could protect the contained material from degradation during information transfer [49]. Thus, we first loaded ATO with hUC-EVs to try to improve the bioavailability of ATO without increasing system toxicity and combined the dual immunomodulatory functions of ATO and hUC-EVs to achieve better efficacy in the present study. We found that ATO was detectable in EVs released from hUC-MSCs after treatment with ATO. Compared with the ATO and hUC-EVs groups, mice in the hUC-EVs-ATO group developed fewer clinical manifestations and lower histological scores of aGVHD, as well as the longest survival in our study, suggesting better therapeutic effects of hUC-EVs-ATO on aGVHD.

Activation and differentiation of donor-derived CD4^+^ T cells are involved in the development of aGVHD [50]. Indeed, the combination of CD4^+^ T cells from the donor and APC cells from the recipient results in the activation of donor T cells and the differentiation of Th cells, which release a variety of cytokines to further mediate the inflammatory response to GVHD. In different cytokine environments, CD4^+^ T cells can differentiate into different subpopulations, including Th1, Th2, Th17 and Treg cells. Th1 and Th17 cells can contribute to aGVHD development, while Tregs can protect against aGVHD [51]. Interestingly, hUC-EVs-ATO reduced the frequency of Th1 and Th17 cells while increasing the Treg cell frequency in vivo but not in vitro. This result suggested that the CD4^+^ T cell immune response in hUC-EVs-ATO-treated mice was not produced by the direct action of hUC-EVs-ATO on T cells but was dependent on other immune cells. Macrophages and T cells can influence each other's phenotypes by secreting different cytokines. M1 macrophages are thought to be proinflammatory cells that can promote the differentiation of Th1 and Th17 cells. M2 macrophages are considered anti-inflammatory cells, resulting in the induction of Tregs [52]. Previous studies have shown that M1 macrophages are predominant in aGVHD and that the M1/M2 ratio is positively correlated with the occurrence of grade 2–4 aGVHD [12–14]. The realignment of the M1/M2 ratio may be beneficial in reducing the severity of aGVHD. Notably, depletion of macrophages blocked the therapeutic effects of hUC-EVs-ATO on aGVHD, which demonstrated that hUC-EVs-ATO may function through macrophages. In support of this notion, we found that the percentages of M1 macrophages (F4/80^+^iNOS^+^ cells) were decreased, while the percentages of M2 macrophages (F4/80^+^CD206^+^ cells) were increased in hUC-EVs-ATO-treated mice. Meanwhile, hUC-EVs-ATO treatment also reduced the levels of the M1-related cytokines TNF-α and IL-1β and increased the levels of the M2-related cytokines TGF-β and IL-10. The switch of M1 to M2 by hUC-EVs-ATO was further confirmed in both BMDM-M1 and RAW264.7-M1 macrophages.

Autophagy, a highly conserved mechanism for self-digestion and recycling of cytoplasmic components, has an important role in the regulation of macrophage polarization. Studies have shown that autophagy is required for macrophages to suppress M1 and promote M2 polarization [53]. Activation of autophagy promotes M1 to M2 phenotypic conversion [54]. Thus, we speculated that hUC-EVs-ATO may regulate the phenotype of macrophages by affecting autophagy. We found a significantly decreased accumulation of P62 and an increased ratio of LC3II/LC3I in both hUC-EVs-ATO-treated mice and BMDMs, which indicated that hUC-EVs-ATO may promote conversion to M2 from M1, possibly by increasing the level of autophagy in M1. The formation of autophagosomes proceeds through three main stages: initiation, elongation and maturation. Activation of the ULK1 complex is required for the initiation of autophagy. It is widely believed that mTOR can inhibit the activation of ULK1, thus negatively regulating autophagy [55]. Previous studies have revealed that sustained activation of mTORC1 inhibited M2 polarization [56]. Therefore, we hypothesized that hUC-EVs-ATO may affect the M1 to M2 transition by regulating the mTOR-autophagy pathway. Our results demonstrated that hUC-EVs-ATO significantly inhibited the expression of p-mTOR in BMDM-M1 macrophages both in vitro and in vivo. Moreover, using an agonist of mTOR (MHY1485) to interfere with BMDM-M1 macrophages, autophagy was inhibited, and the conversion effects of hUC-EVs-ATO on M1- to M2-type macrophages were significantly attenuated, while the effects of hUC-EVs-ATO on conversion to M2 from M1 macrophages were obviously enhanced, when using an inhibitor of mTOR (rapamycin, RAPA). Thus, this study revealed that hUC-EVs-ATO promoted the M1 to M2 conversion of macrophages by inhibiting the mTOR-autophagy pathway. In addition, it should be noted that RAPA has been reported to mitigate GVHD by supporting regulatory T cell expansion [57, 58] and function while inhibiting CD4^+^ helper cells [59], effector CD8^+^ T cells [60] and dendritic cells [61]. Recently, RAPA has also been revealed to promote myeloid-derived suppressor cells (MDSCs), thus attenuating the development of GVHD [62]. Combined with the result in our study that the promoting effects of hUC-EVs-ATO on M2 macrophage polarization were strengthened when macrophages were stimulated with RAPA, it is possible that RAPA may be an attractive approach to strengthen the immunosuppressive function of hUC-EVs-ATO, contributing negatively to GVHD development.

Allo-HSCT benefits these malignancies due to graft-versus-tumor (GVT) effects, and alloreactive effector T cells have been revealed to be critical in GVT effects via cytokine release while mediating aGVHD [63]; thus, it is important and difficult to alleviate aGVHD without weakening GVT effects. In our study, hUC-EVs-ATO were found to reserve GVL effects while reducing aGVHD, indicating that hUC-EVs-ATO are a promising therapeutic approach in aGVHD after allo-HSCT. However, it should be noted that ATO has been shown to inhibit cell proliferation and induce apoptosis in several malignant cell lines, including lymphoma cells [64]; thus, it is currently unclear whether hUC-EVs-ATO reserved GVL effects in our study via direct effects on A20 or via cytokines released by other immune cells, or both. Therefore, future studies will focus on the molecular mechanisms by which hUC-EVs-ATO reduce GVHD while allowing for the maintenance of GVL effects.

Here, we first prepared hUC-EVs-ATO and provided evidence that hUC-EVs-ATO exhibited better therapeutic efficacy in an aGVHD mouse model than hUC-EVs or ATO alone, which was demonstrated to be associated with repolarization of the M1 to the M2 phenotype, and autophagy mediated by mTOR was revealed to be involved in this process in our study. Importantly, hUC-EVs-ATO did not impair GVL activity after transplantation, further indicating that hUC-EVs-ATO could be a potential therapeutic approach in aGVHD after allo-HSCT. However, the effects of hUC-EVs-ATO on the upstream pathway of mTOR and the autophagic metabolic pathway need further exploration. In addition, the specific mechanism by which hUC-EVs-ATO reserved GVL effects should be further studied.

## Conclusion

In this study, we showed that hUC-EVs-ATO could attenuate aGVHD without weakening GVL activity. hUC-EVs-ATO promoted the conversion from M1- to M2-type macrophages by increasing the level of autophagy both in vitro and in vivo. Overall, our findings provided a new approach to reducing aGVHD severity while preserving the beneficial GVL effect.

## Supplementary Information

**Additional file 1.** Supplementary Figures.

**Additional file 2.** All primer sequences used in the qPCR process.

## Data Availability

All data generated or analyzed during this study are included either in this article or in additional files.
