# Correction: hUC-EVs-ATO reduce the severity of acute GVHD by resetting inflammatory macrophages toward the M2 phenotype

**DOI:** 10.1186/s13045-022-01323-2

**Published:** 2022-07-31

**Authors:** Yan Su, Xueyan Sun, Xiao Liu, Qingyuan Qu, Liping Yang, Qi Chen, Fengqi Liu, Yueying Li, Qianfei Wang, Bo Huang, Xiao-Hui Zhang, Xiao-Jun Huang

**Affiliations:** 1 Peking University People’s Hospital, Peking University Institute of Hematology, No. 11 Xizhimen South Street, Xicheng District, Beijing, China; 2 Beijing Key Laboratory of Hematopoietic Stem Cell Transplantation, Beijing, China; 3 Collaborative Innovation Center of Hematology, Peking University, Beijing, China; 4 National Clinical Research Center for Hematologic Disease, Beijing, China; 5 CAS Key Laboratory of Genomic and Precision Medicine, Collaborative Innovation Center of Genetics and Development, Beijing Institute of Genomics, Chinese Academy of Sciences, Beijing, China; 6 Chinese Academy of Sciences, China National Center for Bioinformation, Beijing, China; 7 Department of Immunology, Institute of Basic Medical Sciences, Chinese Academy of Medical Sciences, Beijing, China

## Correction to: Journal of Hematology & Oncology (2022) 15:99 10.1186/s13045-022-01315-2

The original article [1] contained errors in the names of co-authors, Xiao-Jun Huang and Xiao-Hui Zhang which have since been corrected.
