# Supplementary material for: hUC-EVs-ATO reduce the severity of acute GVHD by resetting inflammatory macrophages toward the M2 phenotype
Source: J Hematol Oncol. 2022 Jul 21;15:99. doi: 10.1186/s13045-022-01315-2 (PMC9306027; doi:10.1186/s13045-022-01315-2)
Supplement: Supplementary file 1 — Additional file 1. Supplementary Figures. [file 13045_2022_1315_MOESM1_ESM.pdf]

Supplementary materials

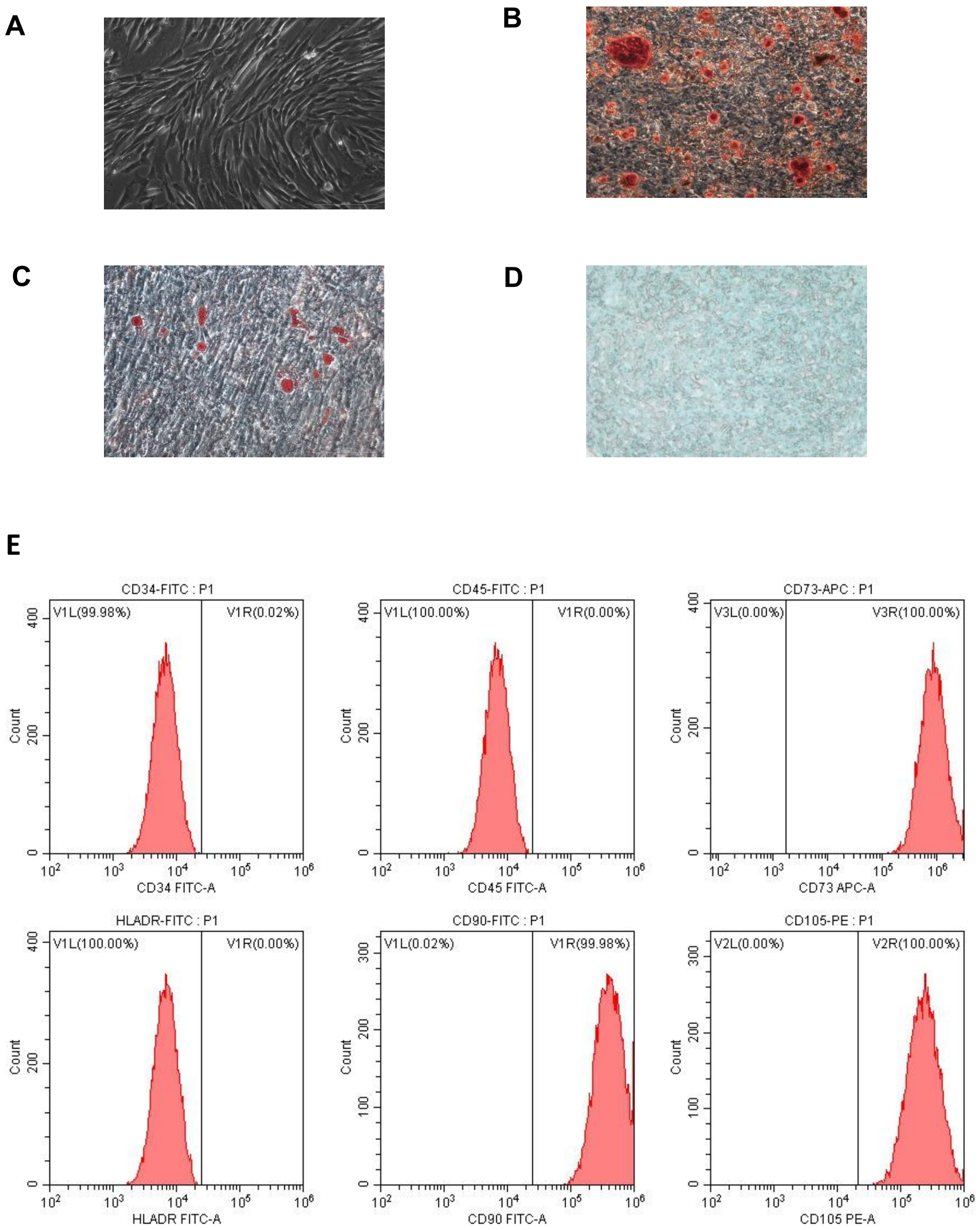

Figure S1. The characteristics of hUC-MSCs. A. Light microscope image of hUC-MSCs. B-D. hUC-HMSCs were examined for differentiation properties by Alizarin Red staining to evaluate osteogenic induction (B) Oil red O staining to evaluate adipogenic induction (C) and Alcian blue staining to evaluate chondrogenetic induction (D). Scale bars are 200μm. (E) Identification of hUC-MSCs via flow cytometry with high expression of CD73, CD90, CD105 and low expression of CD45, CD34 and HLA-DR.

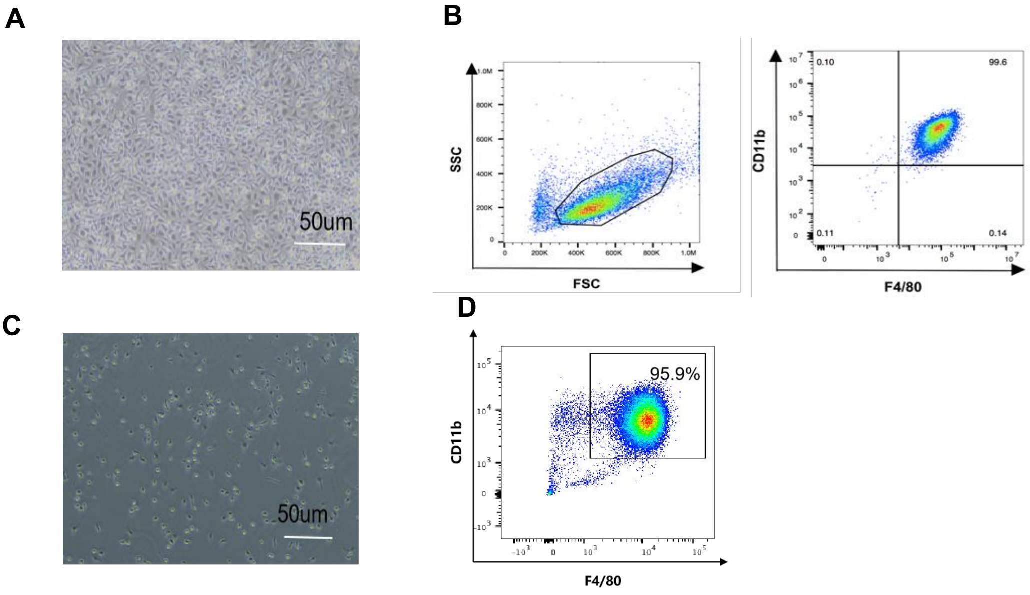

Figure S2. Identification of bone marrow devired macrophage (BMDM) and peritoneal macrophages. Light microscope image and purity of BMDM (A-B) and peritoneal macrophages (C-D). Purity was identified by flow cytometry.

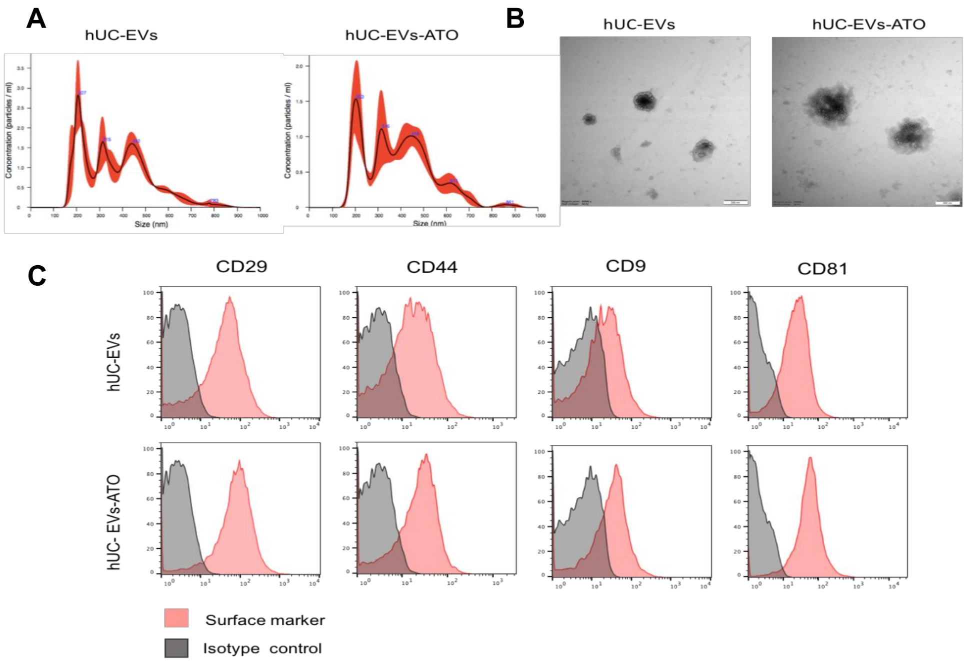

Figure S3.Characterization of hUC-EVs and hUC-EVs-ATO. (A) Particle size distribution by NTA. (B) Morphology by transmission electron microscopy. Scale bar, 200 nm. (C) Expression of surface markers by flow cytometry.

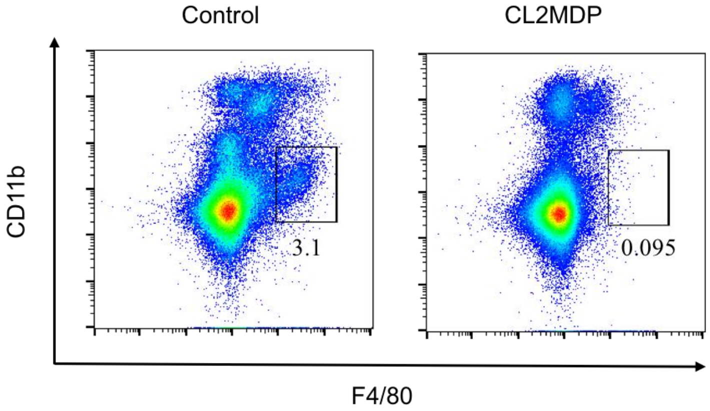

Figure S4. Depletion of macrophages with Cl2MDP liposome. Cl2MDP liposomes were intraperitoneally administrated to mice 72h before injecting hUC-EVs-ATO and once every 3 days to further determine the role of macrophages in hUC-EVs-ATO therapy. Tow days after injection, macrophages in spleen were measured by flow cytometry.

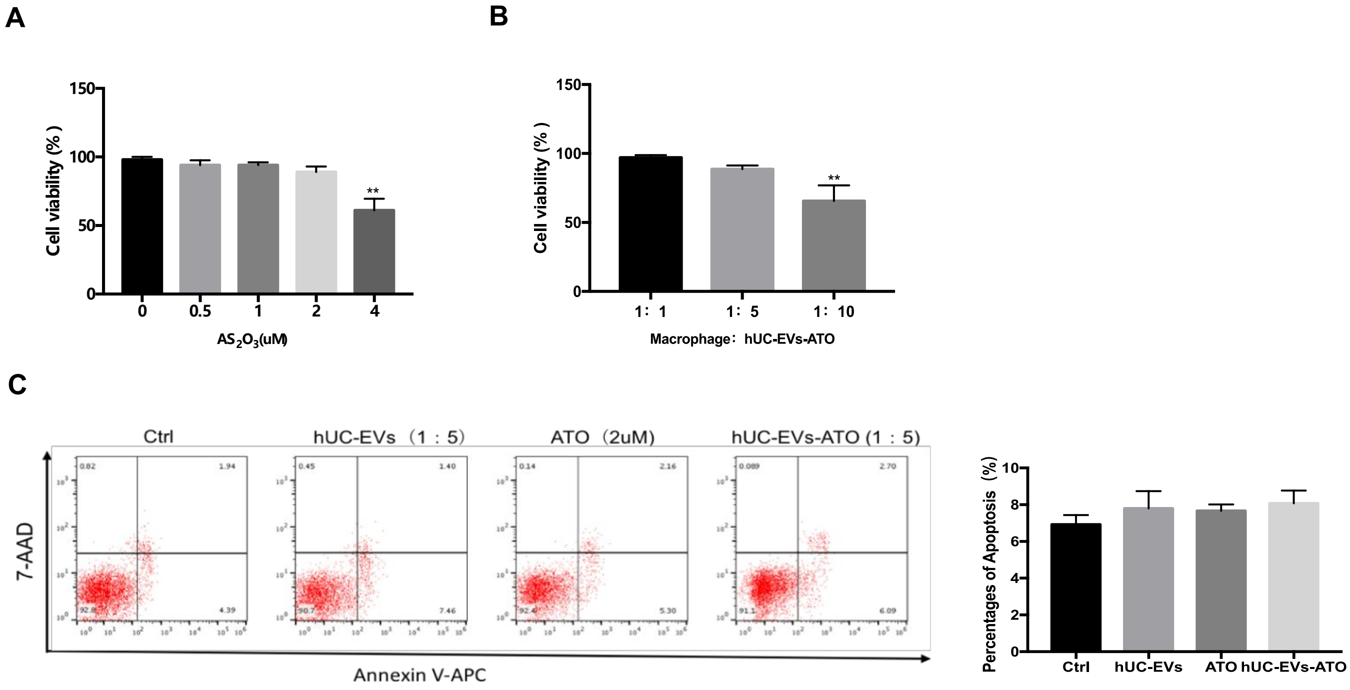

D

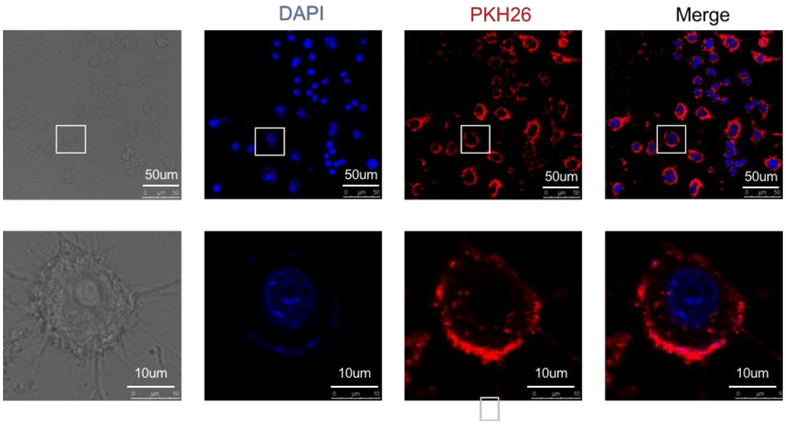
DAPI
PKH26
Merge
50um
10um

E

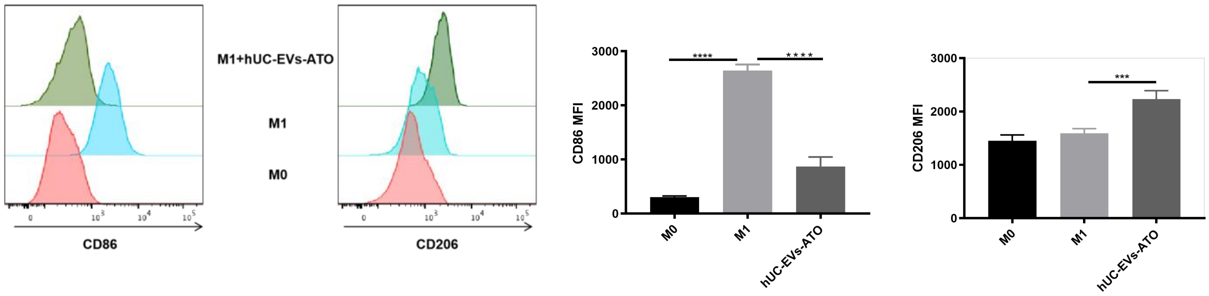
M1+hUC-EVs-ATO
M1
M0
CD86
CD206
CD86 MFI
CD206 MFI
M0
M1
hUC-EVs-ATO
****
***

F

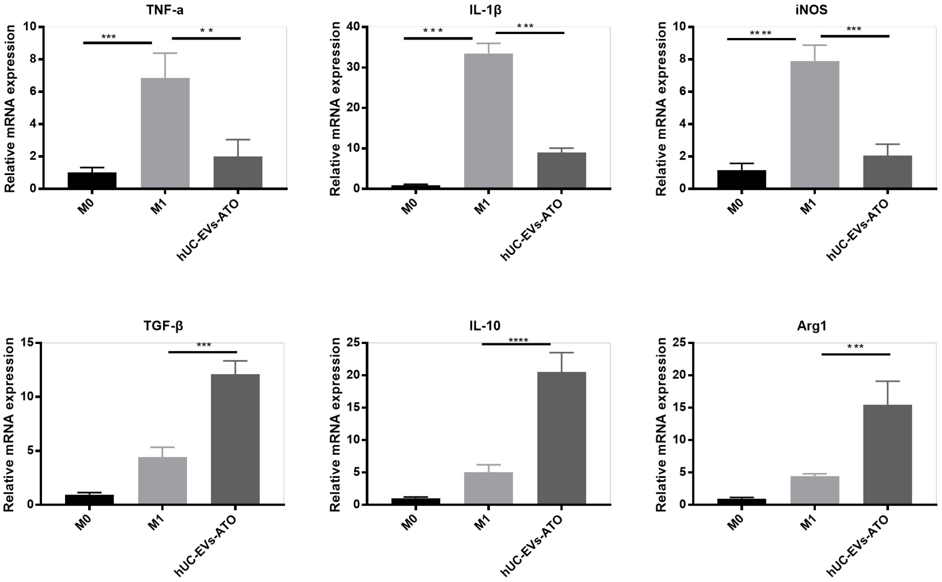
TNF-a
IL-1β
iNOS
TGF-β
IL-10
Arg1
Relative mRNA expression
M0
M1
hUC-EVs-ATO

G

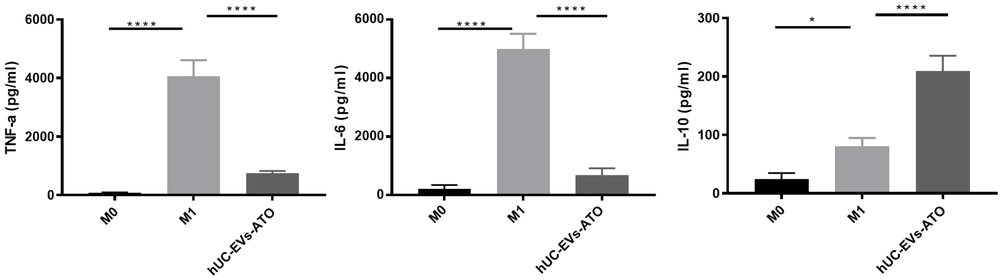
TNF-a (pg/ml)
IL-6 (pg/ml)
IL-10 (pg/ml)
M0
M1
hUC-EVs-ATO

Figure S5. hUC-EVs-ATO could be taken by macrophages RAW264.7 cells and polarized M1 towards M2 in vitro. A. The influence on macrophages viability of ATO with different concentrations via CCK8 assay. B. The influence on macrophages viability of hUC-EVs-ATO with different amounts via CCK8 assay. C. The induction of macropnages apoptosis with ATO or hUC-EVs-ATO. D. hUC-EVs-ATO were labeled with PKH26 (red), and then co-cultured with macrophages for 24h. Nuclei was stained with DAPI. hUC-EVs-ATO was observed in the macrophage membranes and within the cells, reflecting hUC-EVs-ATO could be taken by macrophages. E. Shifting M1 to M2 phenotype under stimulation of hUC-EVs-ATO, with increased expression of CD206 and decreased expression of CD86 by flow cytometry. F. Relative mRNA expression of TNF-a, il-1β,iNOS, TGF-β, IL-10 and arg1 in macrophages under stimulation of hUC-EVs-ATO. Data shown are representative of three independent experiments and presented as mean± SEM. *P < 0.05, **P < 0.01, ***P < 0.001, n.s. = not significant.

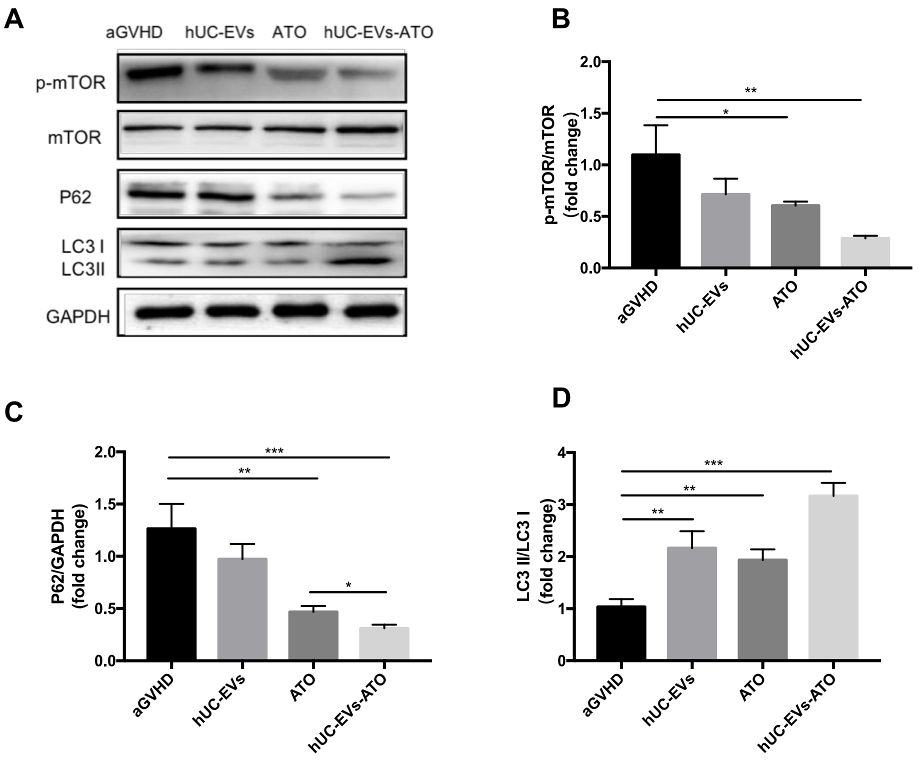

Figure S6. hUC-EVs-ATO affected the mTOR-autophagy pathway of peritoneal macrophages in aGVHD mice. PBS, ATO, hUC-EVs and hUC-EVs-ATO were intraperitoneally injected into aGVHD mice respectively for 5 consecutive days from Day 7 post- transfusion. Collecting peritoneal macrophages in each group at Day 14 after transplantation. A-D. Expression of p-mTOR, m-TOR, P62, LC3 I and LC3 II were assayed in lysates of peritoneal macrophages in each group by Western blotting and were analysed via Image J.
