# Supplementary material for: hUC-EVs-ATO reduce the severity of acute GVHD by resetting inflammatory macrophages toward the M2 phenotype
Source: J Hematol Oncol. 2022 Jul 21;15:99. doi: 10.1186/s13045-022-01315-2 (PMC9306027; doi:10.1186/s13045-022-01315-2)
Supplement: Supplementary file 2 — Additional file 2. All primer sequences used in the qPCR process. [file 13045_2022_1315_MOESM2_ESM.pdf]

Gene	Direction	Primer sequence (5’ to 3’)
TNF-α	Forward	5′- CCTGTAGCCCACGTCGTAG-3′
	Reverse	5′- GGGAGTAGACAAGGTACAACCC -3′
IL-1β	Forward	5′-GAAATGCCACCTTTTGACAGTG -3′
	Reverse	5′-TGGATGCTCTCATCAGGACAG-3′
iNOS	Forward	5′-GGAGTGACGGCAAACATGACT-3′
	Reverse	5′-TCGATGCACAACTGGGTGAAC-3′
TGF-β	Forward	5′ -TCTGCATTGCACTTATGCTGA-3′
	Reverse	5′ -AAAGGGCGATCTAGTGATGGA-3′
Arg1	Forward	5′ -CAAGACAGGGCTCCTTTCAG-3′
	Reverse	5′ -TGGCTTATGGTTACCCTCCC-3′
IL-10	Forward	5′ -AGGCGCTGTCATCGATTTCT-3′
	Reverse	5′ -TGGAGTCCAGCAGACTCAAT-3′
GAPDH	Forward	5′ -TGGCCTTCCGTGTTCCTAC-3′
	Reverse	5′ - GAGTTGCTGTTGAAGTCGCA-3′

Table S1. Primers used for quantitative real time PCR assays.
